# Navigating Workforce Challenges in Long-Term Care: A Co-Design Approach to Solutions

**DOI:** 10.3390/ijerph22040520

**Published:** 2025-03-28

**Authors:** Sheila A. Boamah, Farzana Akter, Bahar Karimi, Farinaz Havaei

**Affiliations:** 1 Faculty of Health Sciences, School of Nursing, McMaster University, Hamilton, ON L8S 4K1, Canada; akterf1@mcmaster.ca; 2 Thrive Group Centre of Excellence in Healthcare Innovation, Hamilton, ON L9C 7N4, Canada; bkarimi@thrivegroup.ca; 3 Faculty of Health, School of Nursing, University of British Columbia, Vancouver, BC V6T 2B5, Canada; farinaz.havaei@ubc.ca

**Keywords:** co-design, mental health, healthcare workers, long-term care, workforce well-being, retention

## Abstract

(1) Background: The enduring impact of COVID-19 on the long-term care (LTC) sector remains uncertain, necessitating targeted efforts to address current and emerging challenges. This study aims to identify the key stressors faced by healthcare workers (HCWs) in LTC and to co-develop innovative, actionable strategies that mitigate these stressors, foster resilience, and promote workforce retention. (2) Methods: This study utilized a qualitative co-design methodology within a mixed-methods, multi-phase framework conducted between July 2023 and October 2024. This article focuses on Phase 1, which involved 11 semi-structured focus groups and steering group discussions with 24 HCWs, including personal support workers (PSWs), nurses, and LTC administrators across Ontario to explore workplace-related distress and foster a shared understanding of challenges in the LTC sector. Data were audio-recorded, transcribed verbatim, and analyzed using thematic analysis to derive key themes and actionable insights. (3) Results: Key themes emerging from co-design sessions included the need for (i) effective workload management tools, (ii) the prioritization of psychological safety and mental health services, (iii) reducing regulatory and bureaucratic burdens, (iv) strengthening management practices, and (v) fostering recognition and a positive sector image. Co-design sessions with HCWs and leaders facilitated the identification of priority issues and high-level solutions, including addressing workload issues, implementing mental health and support programs, enhancing work–life integration, improving management training, and promoting psychological safety in LTC settings. (4) Conclusions: This study deepens our understanding of workplace challenges in the LTC sector and the factors contributing to HCWs’ mental distress. Leveraging a co-design approach offers valuable insights into the lived experiences of HCWs and leaders. The findings provide actionable guidance for LTC leaders and policymakers to create effective, tailored interventions that actively engage HCWs in addressing workplace stressors and mitigating recurrent challenges.

## 1. Introduction

Long-term care (LTC) homes in Canada serve a vital role in supporting vulnerable populations, providing essential care to individuals who require assistance with daily activities and medical needs [1]. Despite their importance, the sector is grappling with persistent challenges, including inadequate funding and resource limitations, which not only compromise the quality of care but also undermine the well-being of healthcare workers (HCWs) who serve in these homes/facilities [1].

The LTC sector in Canada comprises 2076 facilities, including nursing homes, continuing care facilities, and residential care homes, overseen by provincial and territorial governments responsible for their planning, funding, and regulation [2]. Governance within this sector is divided between public (46%) and private (54%) ownership, with the latter further categorized into for-profit (29%) and not-for-profit (23%) entities [2]. While this structure offers diverse care models, it also introduces significant challenges, including system fragmentation, regulatory complexity, chronic understaffing, high resident-to-staff ratios, and an increasingly medically complex resident population [3]. Additionally, disparities in funding and limited public engagement in policy development exacerbate these issues [3]. The structural deficiencies contribute to excessive workloads and difficulties in maintaining consistent, high-quality care, further emphasizing the urgent need for comprehensive systemic reform to better support both HCWs and residents.

As one of the most highly regulated sectors in Canadian healthcare, LTC has faced increasing scrutiny over persistent concerns regarding care quality [4]. However, regulatory oversight, often introduced reactively in response to public scandals and crises, has not necessarily translated into improved accessibility or care standards. This was particularly evident during the COVID-19 pandemic, where LTC homes/facilities bore a disproportionate burden, accounting for over 80% of first-wave deaths—twice the average of other Organisation for Economic Co-operation and Development (OECD) countries [2].

Although concerns about care standards in LTC have been raised for decades [5], the pandemic brought these systemic issues into sharp focus and exposed substantial gaps in the Canadian LTC system, particularly its lack of readiness for public health emergencies. Deficiencies in infection control protocols, restricted access to personal protective equipment (PPE), and inadequate training in infection prevention and control (IPAC) further exacerbated the sector’s vulnerabilities [6]. These shortcomings contributed to significant stress, anxiety, compassion fatigue, and moral distress among HCWs, impairing their mental health and ability to provide high-quality care [7,8,9].

Workplace distress, particularly burnout, is prevalent among HCWs working in high-stress, high-risk environments and is strongly associated with poor mental health outcomes [10,11], reduce attentiveness to residents, increased medical errors, and lower resident and patient satisfaction, all of which impose significant costs [12]. Chronic understaffing further exacerbates these challenges, with 50% of Ontario’s personal support workers (PSWs) leaving the sector within five years, and 43% citing short-staffing as a key reason [13]. Supporting HCWs’ mental health and well-being is vital for their resilience but also essential for improving care quality and achieving better outcomes for LTC residents, reinforcing its importance as a priority for systemic improvement.

While several studies have explored the workplace factors contributing to increased stress and mental health concerns among HCWs broadly [14,15], relatively little research [10,11] has focused on the unique challenges encountered by HCWs in LTC, including PSWs, nurses, and leaders/administrators, both during the pandemic and during its aftermath; and how these issues impact their ability to provide effective resident care. This oversight is acutely critical given the profound impact of COVID-19 on LTC environments, where workers were disproportionately exposed to health risks, emotional strain, and systemic vulnerabilities. The LTC workforce, particularly those in unregulated roles such as PSWs, confronts stark inequities. These workers, who are predominantly women, new immigrants, and individuals from BIPOC communities, remain underpaid, undervalued, and inadequately supported, reflecting systemic disparities within the sector [16]. Compounding this issue is the lack of granular data on the LTC work environment and its effects on worker health and well-being, particularly at the level of individual LTC homes and within specific provincial contexts, further perpetuating these inequities and hindering targeted interventions.

This study seeks to address this critical gap by focusing on HCWs in Ontario’s LTC sector, the most populous province in Canada. By using a co-design approach, it not only delves into the unique needs and challenges faced by these workers—such as factors contributing to distress and burnout—but also actively involves them in developing pragmatic and actionable strategies to improve their well-being and resilience. This approach ensures the study is grounded in the lived experiences of those most affected, paving the way for solutions that are both contextually relevant and impactful.

## 2. Materials and Methods

### 2.1. Study Design

This study utilized a qualitative co-design approach, guided by the Experience-Based Co-Design (EBCD) framework [17], within a mixed-methods, multi-phase design to explore the experiences of HCWs working in LTC homes in Ontario, Canada. This paper focuses exclusively on Phase 1 of the study, as subsequent phases are still in progress.

### 2.2. Co-Design and Its Methodological Benefits

Co-design leverages human-centered design principles to understand experiences and improve services through collaborative, participatory approaches, integrating the creativity of design thinking with systems thinking’s analytical rigor [18]. In healthcare, it effectively engages clinicians and service users to develop interventions that are both feasible and acceptable, particularly in promoting physical activity among older adults [18,19]. While co-design methods vary, they consistently emphasize shared decision-making, ensuring that individuals with lived experience and expertise contribute meaningfully throughout the design process [20,21].

A defining strength of co-design is its commitment to inclusivity, prioritizing marginalized voices and addressing epistemic injustices in traditional decision-making [22]. By critically examining power dynamics, co-design fosters equitable contributions from all stakeholders, leading to more contextually relevant solutions. One specific co-design method, EBCD, exemplifies this approach, integrating narrative-driven engagement with collaborative service improvements [23]. EBCD identifies key “touchpoints” in the patient journey—moments where experiences are most profoundly shaped—to drive meaningful healthcare transformations. Unlike conventional service redesign, which prioritizes process optimization, EBCD focuses on enhancing patient experiences, recognizing their critical role in health outcomes and quality of life.

The increasing adoption of EBCD aligns with the broader shift toward person-centered care and the Institute for Healthcare Improvement’s Triple Aim framework—enhancing patient experience, improving population health, and reducing costs—later expanded to the Quadruple Aim to include provider well-being [24]. Its effectiveness has been demonstrated across diverse healthcare settings, including mental health [25] and elderly care [26], with tangible benefits such as improvements in mental health services and the optimization of medication discontinuation processes for older adults [26]. By embedding patient experiences at the core of service innovation, EBCD cultivates meaningful collaboration between healthcare professionals and service users, ensuring responsive, sustainable, and patient-centered care.

Building on the EBCD framework, this study adopted an adapted approach tailored to the perspectives of HCWs and leaders in LTC, recognizing the potential for transformative impacts on health and well-being. The selection of research methods for exploring HCWs’ and leaders’ experiences was guided by four core EBCD principles [27], as outlined in Text Box 1. In this study, co-design served as a dynamic and promising approach for developing interventions aligned with human-centered principles.

Box 1 **Adapted source: Bate and Robert [27].**

**The initial four stages of EBCD are:**

1. **Observation:** A researcher or non-participant examines the clinical environment to understand user-provider interactions.
2. **Provider interviews:** Interviews with healthcare providers contextualize service delivery and identify key pressure points.
3. **User interviews:** Service users share their experiences, highlighting critical moments in their care journey.
4. **Data analysis:** Emerging shared narratives of defining moments, or touchpoints, capture the essence of both care experiences and workplace dynamics (or work environment).

### 2.3. Sampling, Participants, and Sample Size

This study employed a combination of purposive and convenience sampling strategies to recruit participants from diverse roles within the LTC sector, including PSWs (or care aides), nurses, social workers, managers, and administrators or leaders. To capture a broad spectrum of perspectives and experiences, efforts were made to ensure representation by inviting participants from varied training and professional backgrounds, levels of experience, and geographic locations, including rural and urban settings across Ontario. Emails were sent to various LTC homes and organizations across the province to inform and encourage participation. To promote participation, several webinars and workshops were held. Flyers were distributed across LTC networks, allowing existing contacts to inform others about the opportunity. Social media platforms such as X (formally known as Twitter), LinkedIn, and Facebook were utilized to expand reach and spread recruitment information. All recruitment materials were developed in plain English, employing gender-neutral language and considering sensitivities around ethnicity, race, and intersectional factors to ensure inclusivity. Guided by previous co-design studies [17], the target sample size was 20–25 participants.

Interested participants were required to complete a screening process to verify their work experience in Ontario-based LTC homes and confirm their HCW or leader roles. Eligible participants included HCWs in direct care or leadership roles with at least six months of experience in their current organization and familiarity with the National Standard of Canada for Psychological Health and Safety in the Workplace (“the Standard”) [28]. The Standard provides voluntary guidelines, tools, and resources to support organizations in promoting mental health and preventing psychological harm in the workplace. Potential participants were invited to access the project link to read the letter of information and contact the research team if they were interested in participating in the study. The project team then obtained both written and verbal consent, and participants were informed that participation was voluntary. Participants received an honorarium in the form of a CAD 100 gift card for this phase of the project.

### 2.4. Data Collection

Between July 2023 and October 2024, eleven co-design focus group discussions (FGDs) were conducted over a 10-month period, involving 24 participants. These sessions, held both virtually via Zoom and in person, were organized into two distinct phases. The initial six sessions, with groups of 4–5 participants, explored the work-related experiences of LTC workers, focusing on challenges such as distress and burnout. The subsequent five sessions aimed to collaboratively refine and develop actionable strategies, engaging the same participants throughout. To minimize bias, encourage open and honest dialogue, and mitigate power dynamics, two additional sub-group sessions were organized specifically for certain HCW groups, such as PSWs, to gain insight into their unique needs and challenges in greater depth. Two discussion guides, developed by the research team, facilitated FGDs, which focused on LTC experiences during and after COVID-19. Key themes included workplace conditions, drivers of stress and burnout, and broader social influences on work and personal life. Discussions also covered psychological health, workplace safety, and the evaluation of standard measurement tools, such as the World Health Organization-Five Well-Being Index for assessing mental well-being and identifying distress. Designed as structured consensus-building exercises, the FGDs prioritized outcomes driven by HCWs, aligning with their identified needs and priorities.

Participants were engaged as equal partners in identifying strategies and solutions through a co-design approach that prioritized authentic collaboration. This approach acknowledged and valued the expertise and lived experiences of diverse LTC stakeholders, ensuring their contributions were both integral and central to the process. By fostering inclusivity and shared ownership of outcomes, the approach ensured that the solutions were deeply informed by and authentically reflected the lived realities of those most directly impacted. All FGDs were moderated by a principal investigator—an expert in qualitative interviewing—and supported by a trained facilitator. Open-ended questions encouraged participants to share personal experiences, challenges, strategies, and perceptions of organizational support. Data saturation was achieved when the influx of pertinent new information dwindled or ceased, and any subsequent data only reiterated existing knowledge without enhancing the understanding or contributing additional insights to the established themes [29]. Each session, lasting approximately two hours, was both audio and video recorded for transcription and analysis, with participants’ informed consent. Comprehensive notes, taken by a research team member, supplemented the recordings to ensure accuracy and depth in capturing the discussions.

### 2.5. Data Analysis

The audio from the FGDs was transcribed using Otter.ai, with the research team reviewing and verifying transcriptions for accuracy, noting any inaudible sections. Identifying details were removed to ensure participant anonymity. Transcripts were cross-checked against the audio recordings to resolve discrepancies, ensuring completeness and reliability. Thematic analysis, guided by Braun and Clarke [30], was conducted using Microsoft Excel. The investigator team immersed themselves in the data through repeated listening and reading, with inter-coder reliability checks performed during coding.

The analysis reflected the iterative nature of the co-design method. Initial coding frameworks were informed by research questions and emergent concepts. Two research assistants independently coded each transcript, with discrepancies resolved collaboratively under the guidance of the principal investigator. Preliminary themes were development, capturing challenges, impacts on LTC workers, and potential strategies. Regular team discussions refined these themes, ensuring coherence and responsiveness to the data. A summary of the themes was shared with participants for feedback, which was incorporated to refine the final themes and subthemes. Participants were also given an additional two weeks to provide further verbal or written input. Following principles outlined by Williams and Moser [31], the process emphasized flexibility and depth. Collaborative resolution of ambiguities ensured a rigorous and reflective approach to the analysis.

### 2.6. Ethics Approval and Considerations

Approval for this study was granted by the Institutional Research Ethics Board (blinded for review). In reporting this study, we followed the Consolidated Criteria for Reporting Qualitative Research (COREQ) guidelines [32].

In this research, we critically engaged with the ethical dimensions (ethics of care) of both digital platforms such as Zoom and in-person focus groups by addressing procedural ethics, situational ethics, ethical relationships, and broader ethical concerns [33]. Procedural ethics involved strict adherence to institutional guidelines, including obtaining informed consent, protecting participant confidentiality, and securing institutional ethical approval. Situational ethics required navigating context-specific challenges such as privacy breaches, technical failures, and participant discomfort. Given the heightened privacy risks of digital platforms, the focus groups were conducted in isolated spaces/offices using headphones to minimize unintended exposure—controls more easily managed in face-to-face sessions. Ethical relationships focused on building trust and rapport through intentional engagement, including nonverbal cues like nodding and validating participants’ experiences. Broader ethical concerns encompassed data security, unauthorized access, and digital accessibility. To mitigate cybersecurity risks, we adhered to institutional guidelines, restricting data access to designated team members. Additionally, technical support was provided before and during sessions to address potential digital literacy barriers and ensure equitable participation. While digital platforms introduce unique challenges, ethical considerations—such as obtaining informed consent, maintaining participant confidentiality, and fostering trust—was equally vital in face-to-face interactions. By upholding rigorous ethical standards across both modalities, we ensured a secure, respectful, and equitable research environment.

## 3. Results

A total of 24 HCWs participated in this study, comprising 10 personal support workers (PSWs), 5 registered nurse/registered practical nurses (RN/RPN), 2 social workers (SW), 1 horticultural therapist, 1 behavioral therapist, and 5 administrators/managers (Table 1).

**Figure 1:**
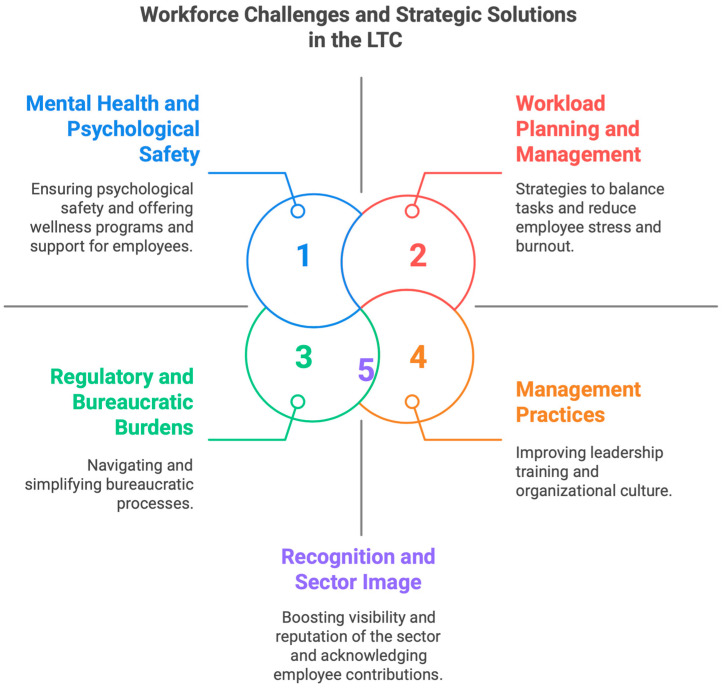
Thematic map of the identified challenges and corresponding solutions.

The findings were organized into five major themes: (i) the need for effective workload management tools, (ii) the prioritization of psychological safety and mental health services, (iii) reducing regulatory and bureaucratic burdens, (iv) strengthening management practices, and (v) fostering recognition and a positive sector image. Each theme reflects the profound impact of the pandemic and its aftermath on workers’ mental, emotional, and professional well-being. The co-design process identified actionable strategies to address these challenges, including sustainable workforce planning, establishing mental health programs, promoting self-care and reducing its stigma, enforcing work–life boundaries, and fostering psychological safety in LTC settings. The findings, organized by theme, with illustrative quotes from participants, provide deeper insight into the lived experiences of LTC workers and are presented in the subsequent sections. Figure 1 illustrates a thematic map of the identified challenges and corresponding solutions.

### 3.1. Identified Needs and Challenges

#### 3.1.1. The Need for Effective Workload Management Tools

The chronic staff shortages during the pandemic increased workloads for HCWs, leading to widespread burnout and exhaustion. Many staff worked extended hours, often exceeding full-time schedules, to cover for absent colleagues. One RN shared the experience, stating,

*“To make up for missed coworkers, many employees put in extra hours, even going above and beyond their full-time schedules. I personally had worked part-time during the pandemic but was working over full-time hours because there just were not enough nurses… The nurses we have were burning out.”*

(Assistant Director of Nursing01)

The heavy workloads were compounded by burnout among PSWs, many of whom took stress leave, time off, or exited the field entirely. One participant noted,

*“Now I find in the PSW staff that they are taking stress leaves, and they are taking, you know, time off and or quitting the field entirely.”*

(Assistant Director of Nursing02)

The mental and emotional toll of these staffing shortages is substantial, with many participants expressing a feeling of helplessness as they attempted to maintain high standards of care under untenable conditions. Participants identified staffing shortages and subsequent heavy workloads and described them as persistent, affecting all levels of staff, from frontline workers to management.

#### 3.1.2. The Prioritization of Psychological Safety and Mental Health Services

The psychological toll on HCWs during the pandemic was immense. Many staff described the experience as emotionally and physically draining, with one participant likening it to “drowning”, stating,

*“It is very much like you’re drowning and I know the frontline felt like that, too.”*

(SW01)

Another added,

*“The staff, I feel, are burnt out and looking to management for some support, but management themselves are burnt out.”*

(Assistant Director of Nursing01)

The quotes vividly depict the overwhelming strain experienced by HCWs during crises. The first highlights the feeling of being overwhelmed and unsupported, likened to drowning, a sentiment shared across frontline workers. The second emphasizes the cascading nature of burnout, where staff seek support from management, only to find that management is equally exhausted, revealing a systemic cycle of stress and exhaustion that hampers effective support and care delivery. The emotional strain was compounded by the need for additional mental health support, as many staff turned to medication for anxiety and depression, as one participant explained:

*“I will tell you; we have had more people than I have ever known before, disclose that they started an anti-anxiety or an anti-depression medication during the course of COVID.”*

(Horticulture Therapist)

Symptoms of post-traumatic stress disorder (PTSD) were also prevalent, as healthcare workers faced high-stakes, high-stress conditions. As one administrator explained,

*“There’s a lot of, there’s a lot of PTSD among a lot of different team members that I’ve seen and experienced, and even personal experience, where, you know, you feel for everything you’ve gone through, and, you know, and if you’re not in long term care, you don’t fully know.”*

(Administrator 01)

Isolation from friends and family further contributed to their distress, with many feeling lonely and disconnected due to the necessary precautions against spreading COVID-19. One participant shared,

*“You start to get isolated from your friends and your family. It’s very sad, actually,”*

(SW02)

Adding to this burden was the stigma around self-care in caregiving roles. Many felt guilty for prioritizing their own well-being, as reflected in the following statement:

*“You’re not allowed to be selfish if you’re a caregiver, but it’s not really selfish. It’s about taking care of yourself.”*

(SW02)

The quote highlights the paradox faced by HCWs in caregiving roles: the cultural and professional expectation that caregivers must prioritize others’ needs above their own often leads to the perception that self-care is “selfish.” However, the participant challenges this notion, emphasizing that self-care is not an act of selfishness but a necessary practice to maintain their well-being and sustain their ability to provide quality care for others. This perspective underscores a critical truth: caregivers cannot effectively support others if they neglect their own physical, mental, and emotional health. It reframes self-care as an essential, rather than indulgent, component of caregiving—a mindset shift crucial to reducing burnout and fostering resilience among HCWs.

#### 3.1.3. Reducing Regulatory and Bureaucratic Burdens

The regulatory and bureaucratic demands placed on HCWs during the pandemic further strained their capacity to provide care. Constant changes in directives from multiple health authorities created confusion and disrupted workflows, leaving staff feeling overwhelmed. One participant described the experience as “being in the vortex” (SW02) due to conflicting requirements from the authorities. The frequent changes in directives also disrupted the efficiency of care delivery. Staff were forced to pivot repeatedly, spending valuable time deciphering and implementing new protocols rather than attending directly to residents’ needs. These constant adjustments created a sense of chaos and left workers feeling disoriented and unsupported:

*“We have both Ministry of Health and Public Health at the same time… it was so insulting and so degrading to have outside people coming in to teach us what to do like we had never been in the sector before.”*

(SW02)

This quote captures the frustration and perceived disrespect felt by LTC staff when external authorities, such as the Ministry of Health and Public Health, intervened during the pandemic with directives and training. It highlights how these actions were perceived as dismissive of their expertise and experience in the sector, undermining their autonomy and competence. This perceived lack of trust in their capabilities added to their emotional burden, leaving many feeling undervalued and unsupported.

#### 3.1.4. Strengthening Management Practices

Management practices during the pandemic often left HCWs feeling unsupported and undervalued. Many participants expressed dissatisfaction with how they were treated, referring to a lack of empathy and prioritization of bureaucratic demands over staff well-being. One participant shared their sense of betrayal, stating,

*“My employer ended up finding a way to push me out of a job I had for 16 years… the new management treats people horribly.”*

(Manager02)

Additionally, the focus on regulatory compliance over employee support further demoralized staff, with one participant noting,

*“I think that we just become numbers… making sure we are compliant with the ministry and all of that… there is some aspect of micromanaging for certain areas of our work, which makes it difficult.”*

(SW02)

This strained the relationships between employees and management, leaving workers feeling unrecognized and overworked.

#### 3.1.5. Fostering Recognition and a Positive Sector Image

Lack of acknowledgment and appreciation for HCWs’ efforts during the pandemic left many feeling demotivated. Despite their sacrifices, participants shared their frustration with the absence of gratitude from management. One participant shared,

*“We were never acknowledged for our knowledge for our work. And it was very, very upsetting.”*

(SW02)

The long-term effects of this lack of recognition were profound, with another stating,

*“There has never, to my knowledge, been any acknowledgement of anything that happened during COVID… none.”*

(SW01)

Another added,

*“You do not have any more time left, and then you are still not appreciated.”*

(Manager02)

This failure to express appreciation or respect for their contributions during a crisis added to the emotional toll and left HCWs feeling abandoned.

On the other hand, the gap between how LTC workers are publicly perceived and the reality of their experiences on the frontlines further intensifies the problem. Participants pointed out that media narratives rarely highlight their successes or the deeply compassionate care they provide. One participant remarked,

*“It’s frustrating that no one talks about how we heal, how we bring people back to life but they just focus on the failures.”*

(Manager01)

The negative perception perpetuated by the media and the lack of internal recognition undermines efforts to foster pride within the sector. It also impacts recruitment and retention, as potential workers see a profession that is underappreciated and poorly supported. Participants suggested a need for consistent and meaningful recognition, both at the organizational and the societal levels. One participant emphasized,

*“The senior leadership needs to acknowledge us more—our efforts, our sacrifices, our humanity. That would make a huge difference. We need more than pizza parties. We need respect, acknowledgment, and appreciation for what we do.”*

(Manager01)

Beyond recognition, participants called for a cultural shift that celebrates LTC as a vital and compassionate field of healthcare. They believe that by amplifying positive stories and recognizing the dedication of its workers, the sector can rebuild trust and pride among both staff and the public.

### 3.2. Proposed Strategies

#### 3.2.1. Workforce Planning

Participants highlighted the urgent need for effective workforce planning to address persistent staffing shortages and workload challenges in LTC. They advocated for proactive measures, including accurate forecasting of staffing needs, targeted recruitment campaigns, and retention strategies centered on competitive wages, comprehensive benefits, and opportunities for professional development. As one participant stated,

*“I think having permanent staff would be a much better solution. Somehow, finding a way to retain staff and new graduates, making long-term care a more attractive option, would help. Right now, a lot of young nurses don’t even want to work here because hospitals pay more.”*

(RPN01)

These initiatives, participants noted, are essential not only to attract new talent but also to retain experienced workers. Furthermore, they highlighted the importance of collaboration among government bodies, regulatory agencies, and LTC stakeholders to establish sustainable funding models that support hiring and retention efforts. By addressing these systemic workforce challenges, participants emphasized, these measures would improve working conditions and the quality of life for HCWs while improving care outcomes for residents.

#### 3.2.2. Mental Health Programs

Structured mental health programs in LTC homes are essential for addressing the psychological challenges, as many participants highlighted the need for concrete mental health measures, such as regular check-ins, access to counselors, and resilience-building workshops. These programs need to normalize discussions around mental health and create an environment where employees feel safe seeking help. As one participant stated,

*“Health and safety might help with the psychological health and safety as a mandated component.”*

(SW01)

Participants also noted that systemic reforms, such as mandating mental health initiatives in workplace policies and incorporating psychological health into national and provincial laws, could ensure emotional and physical support for workers.

#### 3.2.3. Self-Care Promotion

Promoting self-care is a professional need rather than a luxury. Participants acknowledged the critical need for rest and recovery, sharing their strategies for incorporating self-care into their routines. One participant explained,

*“We are really of no use to anybody when we’re not rested, and when we’re not in a good space ourselves.”*

(Behavioral Therapist)

Practices like meditation during lunch breaks or stepping outside for fresh air were highlighted as small but impactful ways to recharge. Managers in LTC homes can actively encourage self-care by training staff to recognize signs of burnout and creating a no-blame culture where workers feel comfortable prioritizing their health. Participants called for structured policies that mandate regular breaks and provide designated quiet spaces for relaxation, which are important to reducing stress and enabling workers to deliver quality care.

#### 3.2.4. Enforcing Work–Life Boundaries

HCWs often struggle with boundaries between work and personal life, ultimately leading to burnout. Participants stressed the need for strict policies to protect personal time and prevent interruptions during vacations or off-hours. Participants also emphasized the importance of setting clear boundaries. Discussion highlighted the importance of effective policies to ensure uninterrupted personal time and organizational efforts to make LTC a more attractive and balanced career option. One participant shared,

*“People like me are not allowed to not be called on vacation or at one in the morning… you have to have a safe space in your personal life to be able to come back.”*

(SW01)

This quote reflects the strain and lack of boundaries faced by some HCWs, highlighting the expectation of constant availability even during personal time. Participants emphasized the need to establish a safe space to recharge, as it is essential for maintaining resilience, well-being, and effectiveness in demanding roles, enabling workers to return with renewed focus and energy.

#### 3.2.5. Fostering Psychological Safety in LTC Settings

Participants highlighted the importance of fostering psychological safety through open communication, collaboration, and mutual trust among team members. Concerns were raised about workplace violence, especially in instances involving cognitively impaired residents who may exhibit behavioral issues. While many expressed empathy toward these residents and their conditions, participants emphasized the need for proactive measures to prioritize safety and well-being for all within LTC environments. One participant remarked,

*“Management needs to understand the mental toll that abuse from residents takes on us. There should be regular check-ins, counseling options, or just an acknowledgment that we need support.”*

(PSW01)

This quote spoke to the need for leadership to recognize the emotional impact of resident-related abuse on HCWs. Participants emphasized the importance of proactive measures, including regular check-ins, access to counseling, and recognition of workers’ challenges, to support their mental well-being. Cultivating an environment where individuals feel safe to voice concerns, share ideas, and learn from mistakes fosters a culture of support, resilience, and continuous improvement in LTC settings. Table 2 presents a summary of the identified challenges and the proposed strategies and solutions.

## 4. Discussion

This study demonstrates the value of employing co-design and active engagement principles to identify challenges faced by HCWs in the LTC sector and to explore practical solutions. By incorporating stakeholder input, this approach enhances the real-world impact of the research. The findings highlight the compounded and persistent challenges confronting HCWs in Canada’s LTC sector, particularly in the aftermath of the COVID-19 pandemic. Consistent with existing literature, these challenges include chronic staffing shortages, psychological and moral distress, and deeply entrenched systemic barriers that impede the delivery of effective care [34,35]. While HCWs and leaders reflected on their current experiences, their narratives were heavily influenced by the enduring impacts of the pandemic, highlighting its profound and lasting repercussions on the sector. Despite the lifting of lockdown measures nearly three years ago, participants emphasized that these challenges remain unresolved, illustrating the ongoing strain on the workforce. This is especially critical in the LTC context, where residents, many of whom belong to vulnerable populations, continue to face heightened risks associated with COVID-19 and its aftermath.

Unsurprisingly, and consistent with other studies [36,37], our findings supported a link between heavy workloads and unmet resident care needs, as well as the significant physical and emotional toll on workers. These challenges were magnified during the pandemic, leading to widespread exhaustion and diminished care quality, further illustrating the critical need for targeted, stakeholder-informed interventions in the sector.

A prominent theme emerging from the co-design discussions was the psychological and moral distress that care workers experienced, an acute issue driven by ethical conflicts, systemic constraints, and the emotional toll of high-stress work environments. Moral distress, which occurs when HCWs are unable to act in accordance with their ethical beliefs due to external constraints, has been linked to long-term psychological impacts such as moral injury, emotional exhaustion, and burnout [38,39]. Similar to the work of Boamah and colleagues [10], LTC workers reported increased workloads, inadequate time for resident care, and limited personal recovery time, further intensifying their moral distress and compounded emotional fatigue.

Our study highlighted systemic barriers—notably, regulatory and bureaucratic burdens—as key contributors to stress among LTC HCWs. Frequent changes in directives from health authorities disrupted workflows and created confusion, echoing broader challenges in the field, where unclear or excessive regulations are linked to heightened stress and inefficiency [40,41,42]. While these demands are crucial for ensuring quality care and accountability, they often impose significant administrative workloads that detract from direct patient care. Combined with chronic understaffing, these burdens intensify workloads, extend shifts, and exacerbate stress, leading to burnout.

Consistent with Ontario’s LTC COVID-19 Commission report [43], participants in this study reported that bureaucratic tasks hinder their ability to provide personalized care, frequently causing moral distress as they balance regulatory compliance with holistic caregiving. Workers described a culture overly focused on rule adherence and over-reporting, often at odds with residents’ health, safety, and well-being. Regulatory frameworks were perceived as overly complex, inflexible, and misaligned with the diverse realities of LTC settings (e.g., urban vs. rural), leaving workers unsupported in adapting to these demands. A prevailing perception of regulatory bodies as adversarial, prioritizing error detection over collaboration, further fueled feelings of scrutiny, fear, and psychological distress. This culture undermines morale, reduces job satisfaction, and underscores the pressing need for regulatory approaches that are more supportive, adaptable, and aligned with both worker and resident needs.

Our findings align with Elstad and Vabø [44], who argued that undervaluation by management and inadequate recognition both at the local and societal levels—along with a lack of appreciation from the media and the public—are factors contributing to job dissatisfaction and turnover intention, particularly in eldercare settings. The demoralizing impact of rigid hierarchies and micromanagement, as highlighted in this study, are consistent with the work by Marcella et al. [45], which underscored the detrimental impact of inflexible organizational structures on staff morale and interprofessional collaboration. These findings illuminate the multifaceted challenges within Canada’s LTC sector, reinforcing the need for targeted interventions to address structural, organizational, and emotional dimensions of HCW well-being.

During the co-design process, HCWs and leaders collaboratively identified several strategies to address the challenges within the LTC sector. Workforce planning emerges as a critical intervention to mitigate chronic staff shortages. Forecasting models project a significant shortfall in PSWs and nurses by 2035, necessitating the urgent need for strategic planning to align workforce supply with future demands [46]. Innovative approaches, such as the program logic model implemented at the Ottawa Hospital, provide actionable solutions for addressing staffing gaps. Recruitment strategies should include attracting unemployed individuals, re-engaging emigrated workers, and recruiting foreign workers (“Ensuring the availability of sufficiently trained carers”) [47]. Furthermore, retention efforts must prioritize improving training programs, developing clear career pathways, and cultivating a positive work culture, as these factors are pivotal to improving job satisfaction and reducing turnover (“Ensuring the availability of sufficiently trained carers”) [47].

Mental health programs and interventions are equally vital. Resilience-building workshops and mindfulness-based interventions were identified as effective measures for mitigating short-term effects of burnout and improving psychological well-being [48,49]. Participants emphasized the need to foster supportive workplace culture that encourages self-care and enforces clear work–life boundaries, thereby protecting staff from further exhaustion and burnout [50,51,52]. Complementary interventions and strategies, such as training HCWs in self-leadership and emotional intelligence, can empower them with the tools needed to manage stress more effectively [53]. As per the study participants, addressing mental health and other systemic issues in LTC necessitates comprehensive structural reforms that go beyond mitigating immediate crises to tackling entrenched issues within the sector.

As highlighted in this study, fostering psychological safety within LTC settings is pivotal for establishing a supportive and collaborative work environment. Lai and Jamal et al. [54,55] emphasize the vital role of leadership in cultivating spaces where staff feel valued and empowered to voice concerns without fear of judgment or reprisal, thereby enhancing both team dynamics and care quality. Interventions such as participatory change management and targeted staff training have been shown to strengthen safety culture and build organizational resilience [56].

HCWs and leaders stressed the need for systemic reforms to improve regulatory practices, streamline administrative processes, and align policies with the practical realities of frontline care. Such measures would better equip and support staff in meeting the complex needs of residents. These findings emphasize the urgent need for collaborative efforts among policymakers, healthcare organizations, and senior LTC leaders/administrators to address systemic challenges. Implementing sustainable reforms is critical not only to enhancing worker well-being but also to improving the overall quality of life within LTC settings.

### 4.1. Implications

The findings suggest that both tangible aspects of care work, such as working conditions, roles, and workplace social relations, and intangible factors, such as perceptions of societal recognition and value, significantly influence staff retention. These results emphasize the need for systemic and organizational reforms within the LTC sector to address the persistent workforce challenges. Priority measures include strategic workforce planning to ensure adequate staffing and prevent burnout, implementing mental health programs or initiatives to support HCWs’ psychological well-being, and fostering a workplace culture that normalizes and promotes self-care. The insights from this study extend beyond the LTC sector, offering valuable guidance for healthcare settings at large and informing policies and practices across diverse jurisdictions.

To achieve meaningful change, policymakers, healthcare organizations, and LTC administrators must prioritize collaborative efforts to allocate resources effectively, streamline regulatory and documentation processes, and implement inclusive workforce strategies. Reducing these regulatory and administrative burdens on non-priority issues aligns with the goals proposed in the Government of Canada’s [57] nursing retention toolkit, focusing on freeing up HCWs to focus on the tasks and care that they are uniquely skilled to provide. Empowering LTC workers through training and flexible guidelines, and involving them in policy-making decisions, can reduce the emotional toll and enhance their ability to deliver quality care.

The findings present actionable strategies to address identified challenges, offering a foundation for future research to implement and evaluate the impact of these interventions on HCWs. By integrating HCWs’ lived experiences into policy and practice, this study advocates for sustainable solutions designed to improve worker satisfaction, retention, and resilience in the LTC sector. Moreover, the co-design approach employed in this study serves as a replicable framework for engaging stakeholders in developing practical and context-sensitive solutions that can be adapted to other high-stress healthcare settings. By actively engaging LTC workers as equal partners in the research process, this approach fostered a collaborative space where participants could candidly share their experiences and priorities, enabling a deeper and more nuanced understanding of the challenges they face compared to traditional qualitative methods [17]. The co-design framework not only highlighted the systemic nature of regulatory burdens but also facilitated the creation of actionable, tailored solutions that reflect the lived realities of those most impacted. This approach effectively bridges the gap between theoretical insights and practical applications, ensuring that proposed interventions are both meaningful and implementable within complex healthcare settings.

### 4.2. Strengths and Limitations

This study’s strengths lie in its innovative use of a co-design approach to actively engage HCWs and leaders in Ontario’s LTC sector. By focusing on identifying challenges and co-developing strategies informed by their lived experiences, the study ensures relevance and applicability to real-world conditions. To our knowledge, no prior workplace interventions in LTC have utilized a co-design methodology to specifically address the needs of HCWs during and after the pandemic, highlighting the novelty of this research. Additionally, the integration of a virtual co-design process, complemented by live discussions, provided significant advantages: it was cost-effective, accessible, and facilitated the inclusion of diverse perspectives. This approach enabled robust collaboration among HCWs and stakeholders, overcoming barriers related to geography and scheduling constraints.

Limitations of this study included participants’ time constraints during the co-design process. Data collection took place during and after the pandemic lockdown (2023–2024), a period when HCWs and leaders were overburdened, making it challenging to secure their participation. To address this, focus group sessions were limited to two hours with scheduled breaks, and asynchronous options, such as email feedback and collaboration tools, were provided for flexibility. However, time constraints may have impacted participation rates, and the range of distress sources identified. Additionally, technological challenges posed risks to engagement, particularly in virtual sessions. To mitigate these issues, a co-host moderator swiftly managed technical difficulties, while staggered participation allowed for flexible involvement at different stages of the design process, accommodating diverse schedules. Future research should expand to include participants from other Canadian provinces and linguistic groups, including Francophones, to enhance representation and applicability.

## 5. Conclusions

The study findings highlighted the profound workforce challenges in Canada’s LTC sector, which has been made worse by the COVID-19 pandemic. The findings reveal the significant impact of short-staffing, moral and psychological suffering, regulatory constraints, ineffective leadership and management practices, and lack of acknowledgment and recognition on the mental health and well-being of HCWs and the quality of care they provide. The study emphasizes that achieving high-quality resident care and fostering a resilient LTC workforce requires prioritizing HCWs’ mental health and improving workplace conditions. Moving forward, a collaborative, multi-stakeholder approach is essential to building a sustainable and supportive LTC environment that benefits both residents and the dedicated individuals who care for them.

## Figures and Tables

**Table 1:** Participant characteristics (*n* = 24).

Profile	*n* (%)
Sex	
Female	21 (87.5)
Male	3 (12.5)
Race	
White	16 (66.7)
Other	8 (33.3)
Role/job title	
Personal support worker (PSW)	10 (41.7)
Nurse (RN/RPN)	5 (20.8)
Leader (director/manager/administrator)	5 (20.8)
Allied health (social worker, behavioral and horticultural therapist)	4 (16.7)
Age range	27–71 years
Mean	50 years
Years of experience	1–30 years

RN = registered nurse, RPN = registered practical nurse.

**Table 2:** Identified challenges and proposed strategies.

Identified Challenges	Proposed Strategies and Solutions
**Workload Management** Chronic staff shortages leading to burnout and exhaustion Extended working hours Stress leave, time off, and attrition among PSWs Recruitment challenges due to non-competitive wages and benefits	**Workload Planning and Management** Workload management tools to distribute tasks equitably, monitor trends, and identify overburdened staff for timely interventions.
**Mental Health and Psychological Safety** Psychological toll, including PTSD, anxiety, and depression Isolation from friends and family Stigma around self-care in caregiving roles Emotional abuse from residents	**Mental Health Program and Promoting Psychological Safety** Access to mental health counselors and resilience-building workshops. Normalizing discussions on mental health and promoting open communication. Promoting self-care as a necessity, with structured policies for breaks and relaxation spaces. Encouraging a no-blame culture and designating quiet spaces for relaxation. Introducing regular check-ins and acknowledgment of mental health challenges by management.
**Regulatory and Bureaucratic Burdens** Conflicting directives from multiple health authorities Perception of external oversight as dismissive and unnecessary	**Streamlined Communication and Policy Coordination** Streamlining communication and collaboration and consolidating directives to minimize confusion. Fostering trust by involving LTC staff in policy discussions and valuing their expertise.
**Management Practices** Lack of empathy and prioritization of bureaucracy over well-being Perception of being treated as numbers rather than valued contributors Micromanagement creating a demoralizing work environment	**Fostering a Supportive and Empathetic Management Culture** Comprehensive training program for management on empathetic leadership and prioritizing staff support alongside compliance. Encouraging managers to actively listen to staff concerns and provide timely, meaningful solutions that prioritize employee well-being. Creating accountability measures to ensure management practices are consistently supportive and aligned with organizational values. Encouraging healthy work–life boundaries be modeled.
**Recognition and Sector Image** Lack of acknowledgment of and appreciation for HCWs’ sacrifices Negative media portrayal undermining sector pride Recruitment and retention challenges due to negative perceptions	**Encouraging Recognition and Elevating the LTC Sector’s Image** Establishing meaningful recognition programs at both the organizational and societal levels. Sharing positive stories and achievements of LTC workers publicly to improve the sector’s image. Highlighting the importance and compassion of LTC roles through media campaigns and professional growth opportunities.

## Data Availability

The study data are not publicly available due to restrictions, such as participant privacy, in line with the ethics agreement. However, data may be accessed upon request by contacting the principal investigator.
